# A G-Quadruplex-Binding Small Molecule and the HDAC Inhibitor SAHA (Vorinostat) Act Synergistically in Gemcitabine-Sensitive and Resistant Pancreatic Cancer Cells †

**DOI:** 10.3390/molecules25225407

**Published:** 2020-11-19

**Authors:** Ahmed Abdullah Ahmed, Stephen Neidle

**Affiliations:** School of Pharmacy, University College London, London WC1N 1AX, UK; a.ahmed.12@alumni.ucl.ac.uk

**Keywords:** quadruplex, CM03, naphthalene diimide derivative, pancreatic cancer, histone deacetylase (HDAC) inhibitor vorinostat, gemcitabine resistance, drug synergy

## Abstract

The stabilisation of G-quadruplexes (G4s) by small-molecule compounds is an effective approach for causing cell growth arrest, followed by cell death. Some of these compounds are currently being developed for the treatment of human cancers. We have previously developed a substituted naphthalene diimide G4-binding molecule (CM03) with selective potency for pancreatic cancer cells, including gemcitabine-resistant cells. We report here that CM03 and the histone deacetylase (HDAC) inhibitor SAHA (suberanilohydroxamic acid) have synergistic effects at concentrations close to and below their individual GI_50_ values, in both gemcitabine-sensitive and resistant pancreatic cancer cell lines. Immunoblot analysis showed elevated levels of γ-H2AX and cleaved PARP proteins upon drug combination treatment, indicating increased levels of DNA damage (double-strand break events: DSBs) and apoptosis induction, respectively. We propose that the mechanism of synergy involves SAHA relaxing condensed chromatin, resulting in higher levels of G4 formation. In turn, CM03 can stabilise a greater number of G4s, leading to the downregulation of more G4-containing genes as well as a higher incidence of DSBs due to torsional strain on DNA and chromatin structure.

## 1. Introduction

Pancreatic cancer is the fourth leading cause of cancer-related deaths in the USA and is expected to be the second or the third in developed and high-income countries in the next ten years [1]. The most common form is Pancreatic Ductal AdenoCarcinoma (PDAC), for which the classic first line standard of care drug in the clinic is the modified nucleoside gemcitabine. This drug and more recently developed adjuvant treatments such as the FOLFIRINOX combination and nab-paclitaxel provide only limited increased overall survival in patients. Five-year survival rates remain low at 8–11% of patients in Europe and USA and at 4–5% for patients with metastatic disease [1]. One reason for poor survival rates is the rapid development of chemoresistance to gemcitabine, which is a major clinical challenge that is associated with more aggressive and metastatic tumours [2].

**Figure 1:**
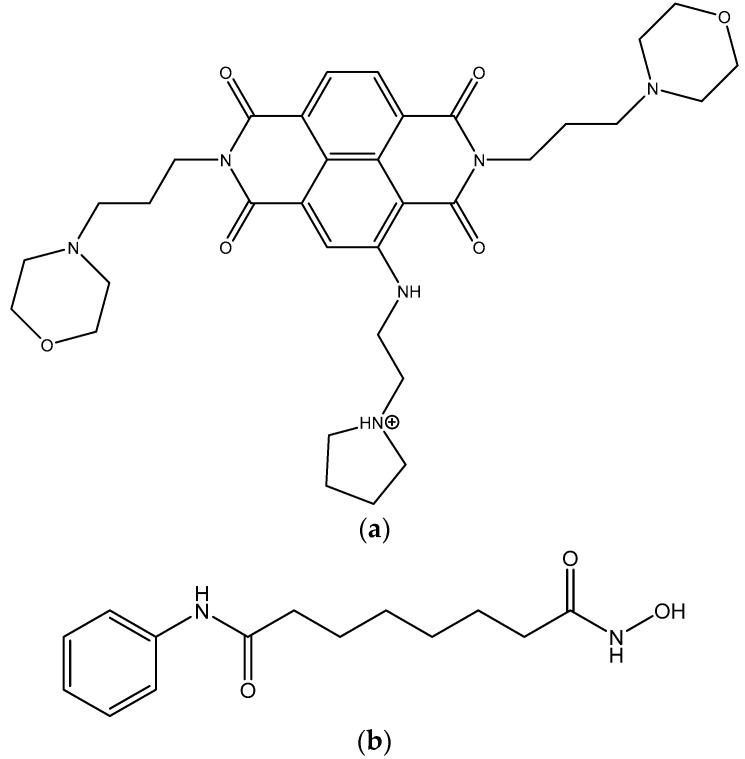
Structure of (**a**) CM03, (**b**) SAHA.

We have recently developed a class of potential drugs for PDAC that are based on the naphthalene diimide (ND) chemotype [3,4]. The lead compounds CM03 and SOP1812 have shown profound in vitro anti-proliferative activity in pancreatic cancer cell lines and significant in vivo anti-tumour activity in several PDAC models, with an improved profile compared to gemcitabine [5,6,7,8,9]. These compounds bind to G-quadruplexes in vitro and in cells, and their mechanism of action is considered to involve the inhibition of cancer gene function by binding to regulatory quadruplex elements in these genes. Compound CM03 (Figure 1a) has also demonstrated sustained efficacy in gemcitabine-resistant PDAC cell lines [10]. Both CM03 and SOP1812 have acceptable pharmacological properties and are currently at preclinical evaluation stages. One approach to their further clinical development may be via synergistic drug combinations that have the potential to widen their therapeutic window and possibly also enhance tumour cell selectivity [11,12].

G-quadruplex (G4) sequences comprising short G-rich repetitive elements can be found in promoter and 5′ and 3′ untranslated regions of many genes (being over-represented in cancer-related genes) and in telomeres [13,14,15,16]. There is also increasing evidence that G4 structures are involved in transcription, translation, and replication, especially of genes involved in human cancer [16,17,18,19,20,21]. The existence of G4s has been demonstrated in live cancer cells [22] and G4-enriched genes have been found to occur in patient-derived breast cancer xenografts [23]. Interestingly, G4s are also directly implicated in epigenetic remodeling via histone modification and DNA methylation regulation during DNA replication [16,17,24,25,26]. This potentially involves the high affinity binding of G4s to epigenetic factors such as DNA methyltransferases (DNMTs) [27]. The stabilization of G4s by small molecules can cause stable epigenetic modifications, which is a more permanent effect than short-term deregulation of transcription and translation [28]. This suggests that G4 ligands can function as epigenetic inhibitors and thus may have potential as a novel drug class for epigenetic targets.

A number of drugs that cause epigenetic remodeling such as methylation inhibitors and histone deacetylase (HDAC) inhibitors are in clinical trials as anticancer drugs [29,30,31], and several (Vorinostat (suberanilohydroxamic acid: SAHA, Figure 1b), romidepsin, and belinostat for T cell lymphomas and panobinostat for multiple myeloma) have received regulatory approval for clinical use. Several HDAC inhibitors (for example, SAHA and panobinostat) are of potential use in the treatment of pancreatic cancer [32,33,34,35,36,37]. Their mode of action involves increasing acetylation by inhibiting histone deacetylase (HDAC) enzymes and other non-histone proteins, resulting in cell cycle arrest and apoptosis [33,36]. SAHA enhances the response to gemcitabine in sensitive PDAC cells when it is given in a drug combination with gemcitabine [37,38] and remains effective in gemcitabine-resistant PDAC cells [2]. In addition, SAHA has low toxicity that makes it and potentially other analogues suitable for drug combination treatment for PDAC [32].

This study has taken as a testable hypothesis the concept that the two effects can act in conjunction and thus synergize each other:

1. Inhibition of chromatin condensation by the HDAC inhibitor SAHA, thus exposing potential G4 sites within a gene, for example in regulatory or non-coding regions.
2. G4-binding compounds can maintain the unwinding of duplex regions of G4-containing genes within active chromatin by stabilizing G4 structures within these genes.

We have used the clinically approved HDAC drug SAHA to investigate potential synergistic effects with a potent G4 binding compound, the naphthalene diimide compound CM03, which was developed in these laboratories [8].

## 2. Results

**Figure 2:**
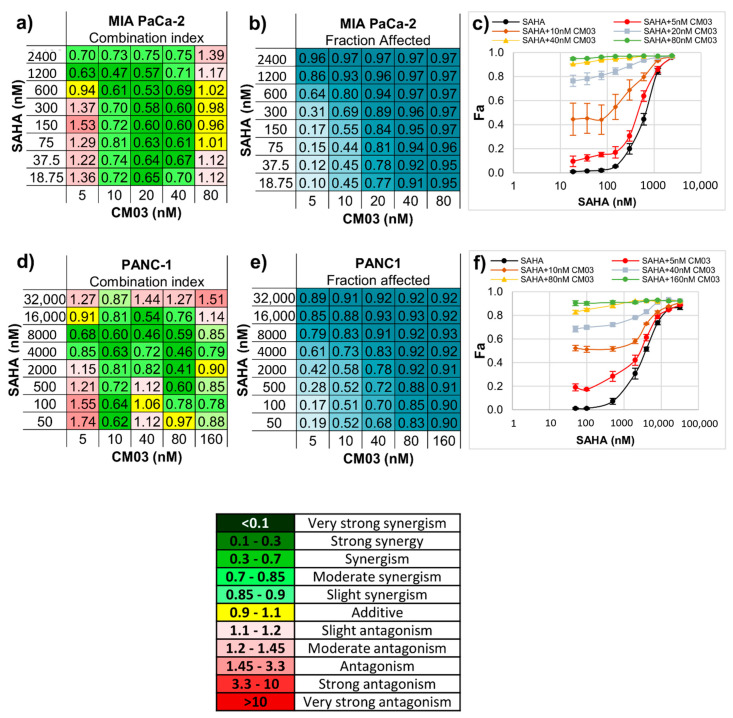
Synergistic effect between CM03 and the HDAC inhibitor SAHA. A scale of the strength of synergy effects is shown. (**a**,**d**) Combination index (CI) values for the drug combination between SAHA and CM03 in (**a**) MIA PaCa-2 and (**d**) PNAC-1 cell lines, CI < 1 indicates synergism (green), CI = 1 additive effect (yellow) and CI > 1 antagonism (red). (**b**,**e**) Fraction affected (Fa) by different combinations of drugs’ concentrations in (**b**) MIA PaCa-2 and (**e**) PNAC-1 cell lines using SRB cytotoxicity assay. (**c**,**f**) Dose–response curves for SAHA alone and in combination with CM03 in (**c**) MIA PaCa-2 and (**f**) PNAC-1 cell lines. Data represents the mean ± SEM of at least three independent experiments.

Synergy between two compounds means that when administered together at lower concentrations than the GI_50_ values of at least one of them, cell growth inhibition of 50% or more is produced. Table 1 details GI_50_ values for the various compounds and drugs used in this study. Figure 2 shows combination index values for a range of concentrations of CM03 and SAHA in MIA PaCa-2 and PANC-1 PDAC cells, together with tables showing the fraction of cells affected at each concentration combination and dose–response curves. In the case of the MIA PaCa-2 cells (Figure 2a–c), there is a spread of concentration combinations showing significant synergy, as judged by combination index values ≤ 0.65 and a fraction of affected cells being ≥0.80. There is a region with a CM03 concentration ≤ 10 nM and where the SAHA concentration is between 600 and 1200 nM where a synergistic effect is apparent and 64–86% of cells are affected. At higher CM03 (10–20 nM) and lower SAHA concentrations (300–600 nM), 69–94% of cells are affected. Synergistic effects are still apparent in PANC-1 cells but are less pronounced (Figure 2d–f). This may be due to the lower sensitivity of PANC-1 cells to SAHA, with a GI50 of ≈4000 nM when compared to MIA PaCa-2 cells with a GI50 of ≈1200 nM (Table 1).

**Figure 3:**
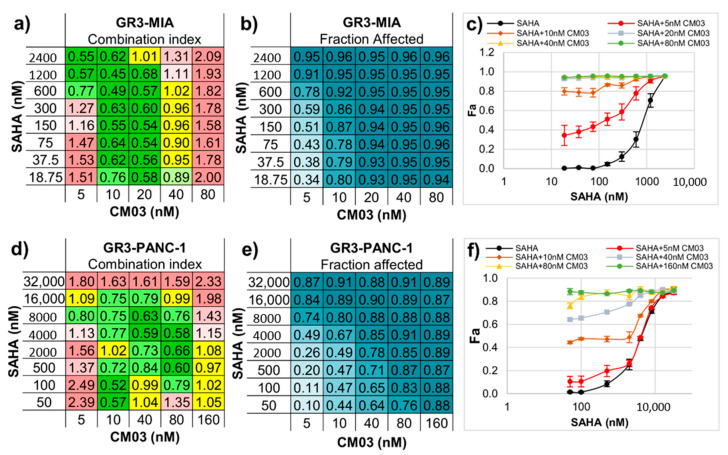
Synergistic effect between CM03 and SAHA in gemcitabine-resistant (GR) Pancreatic Ductal AdenoCarcinoma (PDAC) cell lines. (**a**,**d**) Combination index (CI) values for the drug combination between SAHA and CM03 in (**a**) GR3-MIA and (**d**) GR^3µM^ PNAC-1 cell lines, CI < 1 indicates synergism (green), CI = 1 additive effect (yellow) and CI > 1 antagonism (red). (**b**,**e**) Fraction affected (Fa) by different combinations of drugs’ concentrations in (**b**) GR3-MIA and (**e**) GR^3µM^ PNAC-1 cell lines using SRB cytotoxicity assay. (**c**,**f**) Dose–response curves for SAHA alone and in combination with CM03 in (**c**) MIA PaCa-2 and (**f**) PNAC-1 cell lines. Data represents the mean ± SEM of at least three independent experiments.

Effects in the two gemcitabine-resistant PDAC cell lines are shown in Figure 3a–f. In the GR3-MIA line, there are significant effects (>78% loss of cell viability) at a CM03 concentration of ≤10 nM and SAHA levels down to 18.75 nM and also at 5 nM CM03 and at >300 nM in SAHA, which is well below the GI_50_ value for each individual drug. Less pronounced effects are apparent in the GR3-PANC-1 cell line.

**Figure 4:**
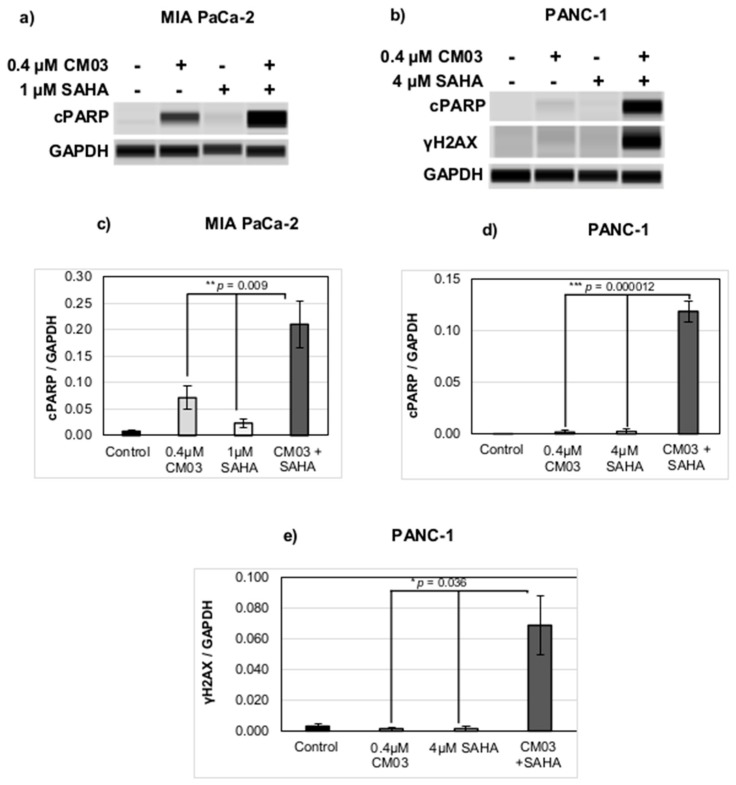
Synergistic induction of apoptosis and DNA damage by the CM03 and SAHA drug combination. (**a**,**b**) Immunoblot analysis of proteins cPARP and γ-H2AX in PDAC cell lines (**a**) MIA PaCa-2 and (**b**) PANC-1, treated with CM03, SAHA, or in combination at the indicated concentrations. (**c**–**e**) Quantification of (**c**,**d**) cleaved PARP (cPARP, apoptosis marker) and (**e**) γH2AX (DNA damage/DSB marker) normalised to the loading control GAPDH in (**c**) MIA PaCa-2 (cPARP only) and in (**d**,**e**) PANC-1. Data represent the mean ± SEM of three independent experiments, and the statistical significance was determined using the One-Way ANOVA test, * *p* < 0.05, ** *p* < 0.01 and *** *p* < 0.001.

The drug combination between SAHA and CM03 also shows a synergistic induction of apoptosis and DNA damage in the wild-type MIA PaCa-2 and PANC-1 cell lines, based on immunoblotting analysis of markers for apoptosis and DNA damage (Figure 4). Higher (though broadly equivalent) concentrations were used in these experiments, with shorter cell exposures than in the combination index experiments. Equivalent results were obtained in both cell lines and show that whereas neither CM03 nor SAHA alone produce significant evidence of apoptosis, as shown by low levels of the PARP apoptosis marker; in combination, the increase in levels of PARP is significant at a >99.9% level, as judged by the one-way ANOVA test. The changes in PANC-1 cells are especially notable, and accordingly, this line was used to examine changes in the levels of the DNA damage marker γ-H2AX. Figure 4e shows that the combination alone produces a statistically significant increase in γ-H2AX levels. 

**Figure 5:**
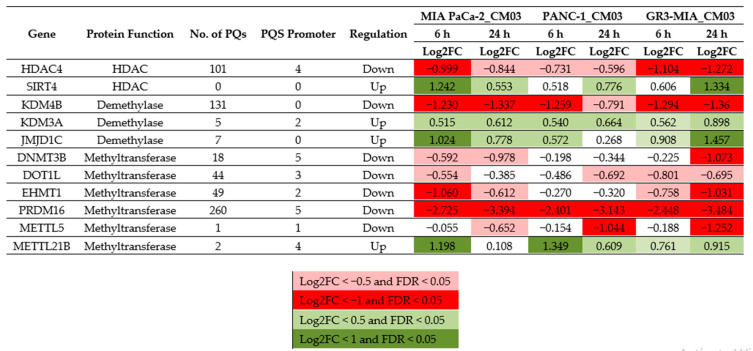
Table of selected epigenetic-related genes and the effects of CM03 on gene expression in pancreatic cancer cell lines. Log2 FC fold changes in gene expression are shown, from RNA-seq analyses. PQs are estimated numbers of putative quadruplex sites. Data taken from [8,10]. Expression changes are grouped in four colour-coded sets, as shown, according to size of change.

Previous studies on CM03 and gemcitabine using RNA-seq methods to map the transcriptome following drug treatment in PDAC cells have identified those genes downregulated by these drugs [8,9,10]. Figure 5 shows effects on a representative panel of those genes especially involved in epigenetic regulation and chromatin reorganisation, including some targets for SAHA e.g., HDAC4, methyltransferases e.g., DNMT3B, PRDM16, and METTL21B and demethylases e.g., KDM4B and JMJD1C. Those genes (notably HDAC4, KDM4B, and PRDM16) with the greatest number of putative quadruplex sites (PQs) are the most downregulated by CM03, consistently in MIA-PaCa2, PANC-1, and the resistant line GR3-MIA. Conversely, those genes with very few PQs show a pattern of consistent upregulation by CM03 (notably SIRT4, JMJD1C, and METTL21B).

## 3. Discussion

The cell-based study reported here has demonstrated that a G-quadruplex ligand (CM03), in combination with the HDAC inhibitor SAHA, can produce >50% synergistic cell growth inhibition in the pancreatic cancer cell lines MIA PaCa-2 and PANC-1, as well as in these derived gemcitabine resistant lines. The study has identified effective two-drug combinations that show these levels of growth inhibition at concentrations below their individual GI50 values. Two other HDAC inhibitors, panobinostat and romidepsin, also show a synergistic effect in combination with CM03 (Supplementary Materials). However, the effects are more profound with SAHA, which could be due to the number of HDACs that can be inhibited by each inhibitor. SAHA is a non-specific HDAC inhibitor and inhibits many classes I, II, and IV HDACs, whereas the other two inhibitors are more discriminating [31,39,40,41]. SAHA does not inhibit class III HDAC enzymes such as the SIRT family. mRNA levels of SIRT4, which can act as a tumor suppressor in pancreatic cancer [42], are upregulated in CM03-treated cells (Figure 5) and are unaffected by SAHA. 

**Figure 6:**
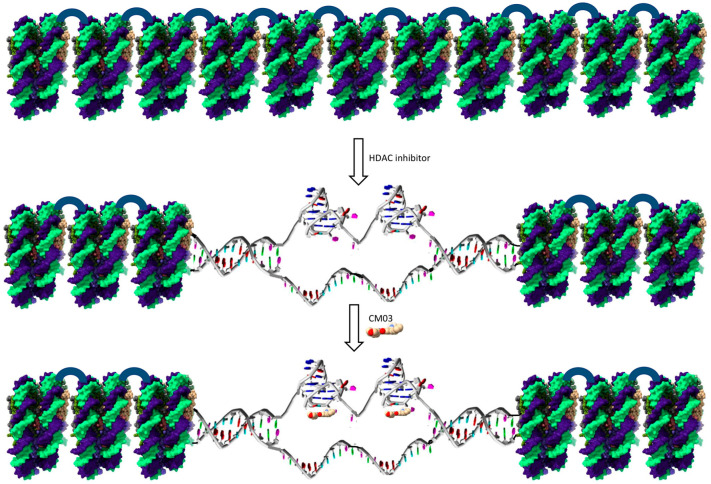
Schematic of effects of CM03 and HDAC inhibitors on chromatin. The top figure shows a stretch of packed nucleosomes. The middle figure shows an effect of the HDAC inhibitor SAHA, inhibiting chromatin remodeling and exposing active chromatin, including genes with G4-forming sequences. The bottom figure shows the stabilization of these G4s by a G4 ligand such as CM03. Then, these stabilized G4-ligand complexes would inhibit the progression of RNA polymerase and lead to inhibition of the transcription of the gene involved.

We propose the following model for the synergistic effect between SAHA and CM03. SAHA, by inhibiting HDACs, induces chromatin relaxation and the formation of euchromatin regions (Figure 6), resulting in more G-quadruplex formation and access to more genes. This effect has been observed in HaCaT cells, using the HDAC inhibitor entinostat and analysis by G4 ChIP-seq, ATAC-seq, and RNA-seq [16]. A large number, >4000 of G4 ChIP–seq sites were found in this study to be in open chromatin regions. We suggest that the quadruplex sites in open chromatin would be stabilized by CM03 binding and thus provide sites for the inhibition of transcription for quadruplex-containing genes. Then, this would lead to growth arrest. Thus, the action of SAHA would be to facilitate the formation of a greater number of quadruplex sites for a given CM03 concentration that would be available with CM03 alone, resulting in growth arrest at lower drug concentrations that with either drug alone. In addition, the induction of quadruplex formation by CM03 would be expected to facilitate chromatin relaxation [24,25,26,27], so augmenting the action of SAHA.

We have previously reported that CM03 in cells produces a modest DNA damage response [8], although other quadruplex binding ligands such as RHSP4 [43], telomestatin [44], and pyridostatin [45] have been well-documented as potent inducers of DNA damage. The significant increase in the level of γ-H2AX protein with the synergistic CM03/SAHA combination is surprising and suggests that the increased chromatin relaxation along with quadruplex stabilization can cause additional torsional strain on the DNA structure, leading to extensive double-strand breaks, which in turn triggers apoptosis [46]. We suggest that this increased DNA damage and apoptosis is a significant contributor to the enhanced cellular growth arrest produced by the drug combination. 

It is also possible that the CM03-mediated downregulation of epigenetic modifiers such as demethylases and methyltransferases can contribute indirectly to the overall synergistic effect by preventing a re-condensation of relaxed chromatin. For instance, PRDM16 is a methyltransferase that maintains the integrity of heterochromatin, and its impairment along with PRDM3 leads to the disintegration of heterochromatic structures [47] Therefore, PRDM16 downregulation by CM03 may also lead to similar effects, aiding the relaxation of chromatin by the HDAC inhibitor SAHA.

This study is the starting point for future more detailed investigations, and therefore, it has some limitations. It is cell-based and has examined only one quadruplex-binding compound. Synergetic effects of CM03 and SAHA have yet to be demonstrated in tumor models in vivo but synergy between quadruplex ligands and other chemotherapeutic agents has been well documented, for example between BRACO19 and paclitaxel [48], cis-platinum with the BRACO19 derivative AS1410 [49], RHPS4 with taxol [50], and the BCL-2 inhibitor Navitoclax with the quadruplex-binding compound GQC-05 [51]. To our knowledge, the present study is the first to report synergy between a quadruplex compound and a chemotherapeutic agent in pancreatic cancer cells, and future studies will extend these to in vivo models for the disease. CM03 alone has in vivo activity but a narrow therapeutic window, which it has in common with several other quadruplex binding compounds [7,8,9,52,53,54]. Although data for in vivo maximum tolerated doses are not always available, a consistent trend for narrow therapeutic windows is apparent (an exception is the substituted perylene compound EMICORON [55]). CM03, with cellular GI_50_ values of 10–15 nM in PDAC cell lines (Table 1), is active in xenograft and genetic PDAC models at biweekly doses of 10–15 mg/kg [8,9]. The present synergy study, with CM03 active in both parental and chemo-resistant cells at concentration down to ca 5 nM and showing cell growth arrest of >50% in synergy with SAHA, suggests that a three-fold reduction of in vivo CM03 dosage to ca 3–5 mg/kg may have a significant anti-tumor effect when in combination with SAHA. This would enlarge the therapeutic window of both compounds, especially CM03, which has been shown to downregulate key genes in PDAC progression [8,9]. 

The present results also suggest that a CM03-SAHA combination is worth considering for human clinical trial evaluation both as a first-line therapy and as second-line therapy for gemcitabine-relapsed (i.e., chemo-resistant) patients. The current clinical choice of FOLFIRINOX (5-fluorouracil plus leucovorin, oxaliplatin, and irinotecan) combination therapy is a demanding therapy and is restricted to those patients with good performance status [56]. Gemcitabine, sometimes with capecitabine, is used as an option for those, especially elderly and other patients who are unable to cope with the toxicity associated with FOLFIRINOX. The CM03-SAHA combination, by contrast, has potential for a low toxicity profile.

## 4. Materials and Methods

### 4.1. Chemicals

CM03 had been previously synthesized and purified as described [8] and was used as the >95% pure hydrochloride/formate salt. Gemcitabine hydrochloride was purchased from Sigma-Aldrich (St. Louis, MO, USA) (Cat. No. G6423). This has a pH of 6.95 in H_2_O. SAHA (Vorinostat, Cat. No. CAY10009929) was obtained from Cayman Chemical Co. (Ann Arbor, MI, USA). It was used without further purification and was dissolved in dimethyl sulfoxide (DMSO). CM03 and gemcitabine were dissolved in phosphate-buffered saline (PBS). Stocks of 1 mM and 10 mM were prepared for each compound and kept frozen and away from light. Prior to addition to cell culture media, compounds were filtered using a 0.22 µm pore-size filter unit to maintain sterility.

### 4.2. Cell Culture

MIA PaCa-2 (Cat. No. CRL-1420) and PANC-1 (Cat. No. CRL-1469) cell lines were obtained from ATCC. Parental and related gemcitabine-resistant cell lines, developed in-house, were all maintained in Dulbecco’s Modified Eagle’s Medium (DMEM) and already contained 2 mM l-glutamine and 4500 mg/L glucose from Sigma-Aldrich (Cat. No. D6429). Media were supplemented with 10% FBS from ThermoFisher (Waltham, MA, USA) (Cat. No. 10270106) and antibiotics 100 U/mL penicillin and 0.1 mg/mL streptomycin from Sigma-Aldrich (Cat. No. P4333). With MIA PaCa-2 and its germ-resistant form, 2.5% horse serum from ThermoFisher (Cat. No. 16050130) was also added. All cell lines were cultured in humidified incubators at 37 °C and 5% CO_2_ and passaged for 2–3 days. The cell lines were routinely tested to ensure that they were mycoplasma-free by a RT-qPCR-based method.

Gemcitabine-resistant cell lines were generated from parental MIA PaCa-2 and PANC-1 pancreatic cancer lines by incrementally increasing the gemcitabine concentration in the culture medium over extended periods of time, as previously described [10]. The resulting resistant cell lines GR3-MIA and GR3-PANC-1, which are both resistant to up to 3.0 µM gemcitabine, were used in this study. The resistant cell lines were continuously maintained in medium containing 3.0 µM gemcitabine, but prior to using them in any experiment, the gemcitabine selection pressure was withdrawn for 7 days to avoid any interference from gemcitabine treatment.

### 4.3. SRB Assays: Synergy Experiments

First, 2000 cells/well of two pancreatic cancer cell lines (MIA PaCa-2 and PANC-1) or their generated gemcitabine-resistant form (GR3-MIA and GR3-PANC-1) were seeded in 96-well plates and incubated overnight. The drug combination SAHA + CM03 was evaluated in the synergy experiments. Cells were treated with two-drug combinations or single drugs for 96 h. A matrix format of 8 × 5 was used to assess all two-drug combinations using a range of concentrations several-fold above below the GI_50_ of each compound. After treatment, cells were fixed with 10% trichloroacetic acid (TCA) and cell viability was measured using a version of the sulforhodamine B (SRB) assay [57]. 

### 4.4. Western Blotting

MIA PaCa-2 and PANC-1 cells were seeded and treated with 0.4 µM CM03, SAHA (1 µM for MIA PaCa-2 and 4 µM for PANC-1) or in combination of both compounds, at these concentrations. The treatment continued for 24 h in MIA PaCa-2 cells and for 48 h in PANC-1 cells because 24 h treatment was insufficient for observing its effect on PANC-1 cells. Untreated or vehicle treated (DMSO) cells were used as a control. Cells were collected and lysed with RIPA lysis buffer (ThermoFisher, Cat. No. 89900) supplemented with protease and phosphatase inhibitors (ThermoFisher, Cat. No. 78442). The lysate concentration was quantified using the Pierce™ BCA Protein Assay Kit (ThermoFisher, Cat. No. 23227). Capillary-based automated Western blotting was performed on a Wes machine (https://www.proteinsimple.com/wes.html) to run the cell lysates and detect proteins of interest according to the manufacturer’s instructions. Antibodies used were for cleaved PARP (Cell Signaling, Cat. No. 5625), γ-H2AX (Novus Biologicals, Cat. No. NB100-384), and GAPDH (Novus Biologicals, Cat. No. NB300-325).

### 4.5. Statistical Analysis 

For the SRB assays, the fraction affected was determined by taking the mean absorbance at 540 nm for each treated cell group expressed as a fraction of the absorbance of untreated or vehicle control-treated (DMSO) control cells. All experiments were performed in triplicate, and the mean ± SEM values were determined from at least three independent experiments. The combination index (CI) values were calculated using the CalcuSyn (version 2.0) software package (https://calcusyn.software.informer.com/ Biosoft, UK) based on the Chou-Talalay non-constant ratio method [58,59]. 

Protein bands were quantified in the immunoblot analysis using the Compass software package (ProteinSimple, San Jose, CA, USA), and the resulting intensity values were normalized to the loading control GAPDH. The one-way ANOVA test was used to determine the statistical significance of any changes in protein levels. Data represents mean ± SEM of 3 independent experiments. * *p* < 0.05, ** *p* < 0.01 and *** *p* < 0.001.

## Figures and Tables

**Table 1:** GI_50_ values (96 h sulforhodamine B (SRB) assay) for the drugs used in this study (CM03, gemcitabine, and histone deacetylase (HDAC) inhibitor SAHA (suberanilohydroxamic acid)).

Compound	MIA PaCa-2 Parental	MIA PaCa-2 GemResist 3 µM	PANC-1 Parental	PANC-1 GemResist 3 µM
**Gemcitabine**	6.5 ± 0.7	11,055.7 ± 540.0	23.3 ± 8.4	28,750.9 ± 6121.3
**CM03**	13.0 ± 8.4	14.9 ± 8.3	10.4 ± 1.2	15.5 ± 1.8
**SAHA**	1292.7 ± 204.5	1055.9 ± 80.5	3986.5 ± 646.6	4499.3 ± 932.7

## Supplementary Materials

The following is available online: Supplementary Figure: Synergistic effect between CM03 and the HDAC inhibitors panobinostat and romidepsin in MIA PaCa-2 cells.
