# Supplementary material for: A G-Quadruplex-Binding Small Molecule and the HDAC Inhibitor SAHA (Vorinostat) Act Synergistically in Gemcitabine-Sensitive and Resistant Pancreatic Cancer Cells
Source: Molecules. 2020 Nov 19;25(22):5407. doi: 10.3390/molecules25225407 (PMC7699281; doi:10.3390/molecules25225407)
Supplement: Supplementary file 1 [file molecules-25-05407-s001.pdf]

Supplementary Data

A G-quadruplex-binding small molecule and the HDAC inhibitor SAHA (Vorinostat) act synergistically in gemcitabine sensitive and resistant pancreatic cancer cells [†]

Ahmed Abdullah Ahmed and Stephen Neidle

Supplementary Figure. Synergistic effect between CM03 and the HDAC inhibitors panobinostat and romidepsin in MIA PaCa-2 cells.

(a,d) Combination index (CI) values for the two drug combinations a) CM03 + panobinostat and d) CM03 + romidepsin, CI < 1 indicates synergism (green), CI = 1 additive effect (yellow) and CI > 1 antagonism (red).

(b,e) Fraction affected (Fa) by different combinations of drug concentrations using the SRB cytotoxicity assay.

(c,f) Dose-response curves for HDAC inhibitor alone and in combination with CM03. Data represents the mean ± SEM of at least three independent experiments.

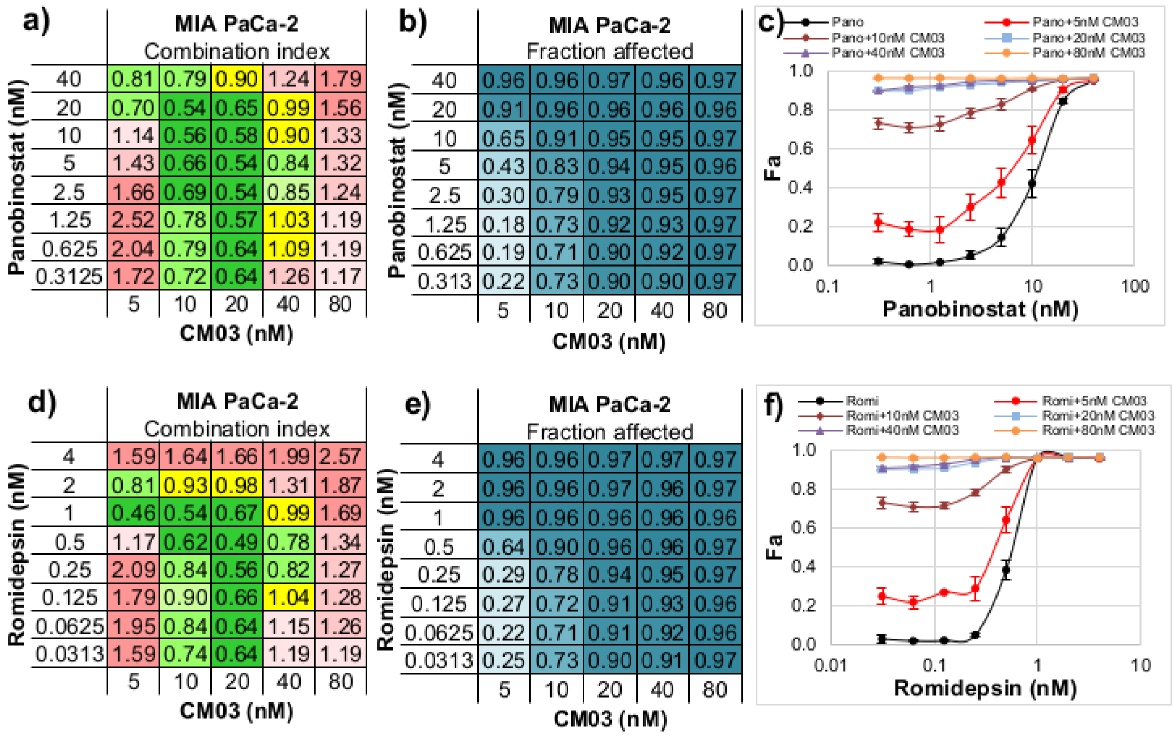

a) MIA PaCa-2 Combination index

Panobinostat (nM) \ CM03 (nM)	5	10	20	40	80
40	0.81	0.79	0.90	1.24	1.79
20	0.70	0.54	0.65	0.99	1.56
10	1.14	0.56	0.58	0.90	1.33
5	1.43	0.66	0.54	0.84	1.32
2.5	1.66	0.69	0.54	0.85	1.24
1.25	2.52	0.78	0.57	1.03	1.19
0.625	2.04	0.79	0.64	1.09	1.19
0.3125	1.72	0.72	0.64	1.26	1.17

b) MIA PaCa-2 Fraction affected

Panobinostat (nM) \ CM03 (nM)	5	10	20	40	80
40	0.96	0.96	0.97	0.96	0.97
20	0.91	0.96	0.96	0.96	0.96
10	0.65	0.91	0.95	0.95	0.97
5	0.43	0.83	0.94	0.95	0.96
2.5	0.30	0.79	0.93	0.95	0.97
1.25	0.18	0.73	0.92	0.93	0.97
0.625	0.19	0.71	0.90	0.92	0.97
0.313	0.22	0.73	0.90	0.90	0.97

d) MIA PaCa-2 Combination index

Romidepsin (nM) \ CM03 (nM)	5	10	20	40	80
4	1.59	1.64	1.66	1.99	2.57
2	0.81	0.93	0.98	1.31	1.87
1	0.46	0.54	0.67	0.99	1.69
0.5	1.17	0.62	0.49	0.78	1.34
0.25	2.09	0.84	0.56	0.82	1.27
0.125	1.79	0.90	0.66	1.04	1.28
0.0625	1.95	0.84	0.64	1.15	1.26
0.0313	1.59	0.74	0.64	1.19	1.19

e) MIA PaCa-2 Fraction affected

Romidepsin (nM) \ CM03 (nM)	5	10	20	40	80
4	0.96	0.96	0.97	0.97	0.97
2	0.96	0.96	0.97	0.96	0.97
1	0.96	0.96	0.96	0.96	0.96
0.5	0.64	0.90	0.96	0.96	0.97
0.25	0.29	0.78	0.94	0.95	0.97
0.125	0.27	0.72	0.91	0.93	0.96
0.0625	0.22	0.71	0.91	0.92	0.96
0.0313	0.25	0.73	0.90	0.91	0.97
